# Gender differences in the association between socioeconomic status and hypertension incidence: the Korean Genome and Epidemiology Study (KoGES)

**DOI:** 10.1186/s12889-015-2175-6

**Published:** 2015-09-03

**Authors:** Tae-Hwa Baek, Hae-Young Lee, Nam-Kyoo Lim, Hyun-Young Park

**Affiliations:** Division of Cardiovascular and Rare Disease, Center for Biomedical Science, Korea National Institute of Health, Cheongju, 187 OsongSaengmyeong2-Ro, Osong-Eup, Cheongju, Chungcheongbuk-Do 361-951 Republic of Korea; Department Internal Medicine, Seoul National University Hospital, Seoul National University College of Medicine, Seoul, Republic of Korea

## Abstract

**Background:**

Hypertension is a leading cause of cardiovascular events. We examined whether there was a gender difference in the association between SES, measured by education and income, and hypertension incidence.

**Methods:**

Data for 2596 men and 2686 women aged 40–69 years without hypertension at baseline from the Korean Genome and Epidemiology Study (KoGES) were analyzed. Participants had two follow-up examinations during 4 years, and were classified into three categories by self-reported educational attainment: ≥ 10 years, 7–9 years, and 0–6 years, and monthly household income (×10,000 Korean Won): ≥ 200, 100–199, and <100. The association between SES and incidence hypertension was examined by Cox’s proportional hazard regression analyses.

**Results:**

Adjusting for conventional risk factors, compared with the high education group (reference), the hazard ratios (95 % confidence interval) for incident hypertension across the education categories were 1.54 (1.16–2.06) and 1.80 (1.36–2.38) in women and 1.15 (0.92–1.43), and 1.08 (0.84–1.38) in men. Women with the low household income were more likely to have hypertension than those with the high household income and incident hypertension had an inverse association with household income level in women: multivariate adjusted hazard ratios were 1.00 (reference), 1.10 (0.83–1.45), and 1.63 (0.75–2.16). Men with medium income were less likely to have hypertension compared with those with high income (0.76, 0.61–0.90).

**Conclusions:**

Educational level and economic status had stronger impacts on hypertension in Korean women than men. Thus, a stratified approach for women of low socioeconomic status, especially those with low educational attainment, is needed for the prevention of hypertension.

## Background

Hypertension is an important risk factor for cardiovascular disease [1]. Globally, 16.5 % of all deaths can be attributed to high blood pressure and this includes 51 % and 45 % of deaths due to stroke and coronary heart disease, respectively [2]. Additionally, hypertension is a leading risk factor for global disease burden in 2010 [2]. The ‘conventional’ risk factors for hypertension include age, obesity, physical inactivity, excess sodium intake, and excess alcohol consumption. However, these known factors do not fully explain the development of hypertension.

Recently, there has been considerable evidence linking socioeconomic status (SES) with hypertension and its associated risk factors [3–6]. Several authors have reported a strong association between SES and hypertension; however, the vast majority of these studies have cross-sectional designs [7–9], whereas data from prospective studies are scarce [10–12]. In the Korean population, very few studies have been conducted, and with conflicting results [13–15]. To our knowledge, no prospective study has been reported on the relationship between SES and hypertension incidence. Moreover, gender differences between SES and the risk of hypertension in large populations of men and women have not been well established in Korea.

Thus, the aims of the study were to examine whether there is a gender difference in the association between SES, determined by educational attainment or monthly household income, and hypertension incidence using community-based cohort data and, if so, to evaluate whether adiposity, one of the main risk factors for hypertension, mediates the relationship between SES and hypertension incidence and is a contributor to the gender difference.

## Methods

### Study participants

The Korean Genome and Epidemiology Study (KoGES) is an ongoing, prospective, community-based cohort study that began in 2001. The aim of the KoGES is to examine relationships between genetic, environmental, and lifestyle determinants associated with chronic diseases, such as diabetes mellitus, cerebrovascular disease, and hypertension, in Koreans. Participants included residents of urban (Ansan) and rural (Ansung) areas. The enrollment of the study was based on the characteristics of the community and on the efficient method for recruiting representative samples of the Korean population. Initially, the cohort included 10,038 participants (5020 and 5018 from the Ansan and Ansung areas, respectively) aged 40–69 years between 2001 and 2003. Follow-up examinations are conducted biennially. This study was based on the baseline and second (year 4) follow-up data.

The study protocol was approved by the Institutional Review Board of the Korea Centers for Disease Control and Prevention. Written informed consent was obtained from all participants.

**Fig. 1:**
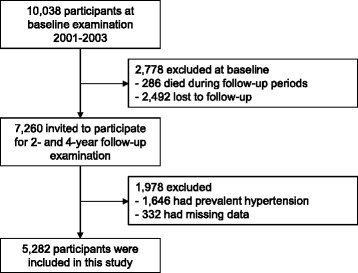
Study flow and participants at baseline and follow-up

Of the original 10,038 participants, 2492 refused to participate in the follow-up surveys and 286 died prior to completing two follow-up visits. Thus, after the 4-year follow-up examination, 7260 participants were enrolled in the study. In addition, those with hypertension (*n* = 1646) and missing covariates (*n* = 332) were excluded. Thus, 5282 participants remained eligible for analysis (Fig. 1).

### Data collection

A standard questionnaire was used to obtain information on demographic characteristics, socioeconomic status, medical history, smoking status, and alcohol consumption at baseline. The study protocol and questionnaire were standardized after assessing the inter- and inta-validity of questionnaire and interviewers during the preliminary examination in 2001. Socioeconomic status included educational attainment and monthly household income. Educational attainment was categorized into three groups: less than 7 years (elementary school graduates), 7–9 years (middle school graduates) and, more than 10 years (high school graduates). Monthly household income was also categorized into three groups: less than 1,000,000 KRW (≈US$1000 at 2014), 1,000,000–1,999,999 KRW (≈US$1000–1999) and 2,000,000 KRW or more (≈US$2000). Current smoking status was defined as smoking at least 1 cigarette per day for at least 1 year. Significant alcohol consumption was defined as ≥ 30 g per day for men and ≥ 20 g per day for women.

Anthropometric measurements were performed by trained healthcare providers. Height and body weight were measured with the participants barefoot and wearing light clothing. Waist circumference (WC) was measured at the midpoint between the lower rib margin and the top of the iliac crest in the standing position. Body mass index (BMI) was calculated by dividing the weight in kilograms by the height in meters squared.

Blood pressure was measured by trained healthcare providers using mercury sphygmomanometers (Baumanometer-Standby; W.A. Baum Co. Inc., New York, NY). Systolic blood pressure (SBP) and diastolic blood pressure (DBP) were defined as the average of the both arm readings obtained after 5 min of rest in a seated position with arms supported at the level of the heart.

### Definitions of obesity, central obesity, and hypertension

According to International Obesity Task Force (IOTF) and the World Health Organization (WHO) Regional Office for the Western Pacific Region guidelines [16], obesity was defined as BMI ≥ 25 kg/m^2^. Central obesity was defined as WC ≥ 90 cm for men and ≥ 85 cm for women, according to the Korean Society for the Study of Obesity (KSSO) guidelines [17]. Hypertension was defined as SBP ≥ 140 mmHg or DBP ≥ 90 mmHg, or self-reported use of antihypertensive medication at the baseline examination.

### Statistical analyses

Data analyses were conducted using the SAS software (ver. 9.2; SAS Institute, Cary, NC, USA) and were undertaken for men and women separately. The main outcome was the cumulative incidence of hypertension at the 4-year follow-up. Basic characteristics of the study population were described. Continuous variables are expressed as means ± standard deviations (SD), and categorical variables are expressed as numbers (percentage). T-tests were used to compare means of continuous variables, and the *χ*^2^ test was used to compare proportions of categorical variables. To assess the association between educational attainment or household income and incidence of hypertension, Cox’s proportional hazard regression analyses were performed after adjusting for potential confounders. Model 1 adjusted for age, smoking status, alcohol consumption, blood pressure level, and family history of hypertension. Model 2 adjusted for the model 1 variables with additional adjustment for BMI. Model 3 adjusted for the model 1 variables with additional adjustment for WC. Additionally, to determine the effect of adiposity (obesity or central obesity) on gender difference in incidence of hypertension by SES, another Cox’s proportional hazard regression model in different strata of BMI (≥25 or <25) and WC (central obesity or not) was conducted. In this model, confounders were adjusted, as described for model 1. For each analysis, tests for linear trends across educational attainment categories or monthly income categories were performed. Hazard ratios (HRs) and 95 % confidence intervals (CIs) were estimated. All tests were two-tailed, and *p*-values < 0.05 were considered to indicate statistical significance.

## Results

Data from a total of 5282 individuals (2596 men, 2686 women) were analyzed. The cumulative incidence of hypertension was 18.4 % (971 cases) at the 4-year follow-up. Table 1 shows the baseline characteristics of the study population stratified by gender. The mean age (SD) was 50.9 (8.5) years for men and 51.0 (8.6) years for women. The mean SBP, DBP, and waist circumference were significantly higher in men, while BMI was higher in women. Overall, about two-thirds of the men had 10–12 years of education (38.3 %) or 13 or more years of education (22.8 %), whereas about two-thirds of the women had 7-9 years of education (24.1 %) or 6 or less years of education (38.8 %). The proportion of individuals with a high monthly income was higher in men than women. Approximately half of the men reported current smoking (48.7 %) and significant consumption of alcohol (31.5 %), compared with only 3.5 % and 1.5 % of women. A positive family history of hypertension was higher in women.

**Table 1:** Baseline characteristics of study population

Variables	All (*n* = 5282)	Subgroup		
		Men (*n* = 2596)	Women (*n* = 2686)	*P* value
Age	51.0 ± 8.5	50.9 ± 8.5	51.0 ± 8.6	0.669
40–49	2828 (53.1)	1401 (54.0)	1427 (53.1)	0.672
50–59	1341 (25.3)	661 (25.5)	680 (25.3)	
60–69	1113 (21.6)	534 (20.6)	579 (21.6)	
SBP	111.1 ± 12.4	111.9 ± 11.7	110.2 ± 13.0	<0.001
DBP	71.7 ± 9.0	73.1 ± 8.9	70.3 ± 9.0	<0.001
BMI	24.3 ± 3.0	24.1 ± 2.9	24.5 ± 3.2	<0.001
WC	81.8 ± 8.6	83.0 ± 7.5	80.6 ± 9.4	<0.001
Education (years)				
High (≥10)	2583 (48.9)	1586 (61.1)	997 (37.1)	<0.001
Medium (7–9)	1217 (23.0)	569 (21.9)	648 (24.1)	
Low (0–6)	1482 (28.1)	441 (17.0)	1041 (38.8)	
Monthly income (*10^4^ KRW)				
High (≥200)	2062 (39.0)	1144 (44.07)	918 (34.18)	<0.001
Medium (100–199)	1568 (29.7)	791 (30.5)	777 (28.9)	
Low (<100)	1652 (31.3)	661 (25.5)	991 (36.9)	
Family history of hypertension				
No	4352 (82.4)	2174 (83.7)	2178 (81.1)	0.011
Yes	930 (17.6)	422 (16.3)	508 (18.9)	
Current smoking status				
No	3931 (74.4)	1332 (51.3)	2599 (96.8)	<0.001
Yes	1351 (25.6)	1264 (48.7)	87 (3.2)	
Alcohol consumption				
No	4425 (83.8)	1779 (68.5)	2646 (98.5)	<0.001
Yes	857 (16.2)	817 (31.5)	40 (1.5)	

Data are presented as mean ± SD or number (percentage). Comparisons performed with independent two-sample *t*-test for continuous variables and with *χ* ^2^-test for categorical variables. *SBP* systolic blood pressure; *DBP* diastolic blood pressure; *BMI* body mass index; *WC* waist circumference

Table 2 shows the HRs for incident hypertension according to each category of monthly income and education attainment. In women, the incidence of hypertension showed a significant inverse association with education level. Individuals with medium (7–9 years) and the low (≤6 years) education levels were 1.54-fold and 1.80-fold more likely than those with the high (≥10 years) education level (reference group) to be hypertensive after adjusting for age, smoking status, alcohol consumption, blood pressure level and family history of hypertension (Model 1). The associations were slightly reduced but remained significant after additional adjustment for BMI (Model 2: HR = 1.52, 95 % CI = 1.14-2.03 for medium education level and HR = 1.75, 95 % CI = 1.32-2.31 for the low education level) or WC (Model 3: HR = 1.49, 95 % CI = 1.12-1.99 for medium education level and HR = 1.67, 95 % CI = 1.26-2.21 for the low education level). In men, there was no significant relationship between incident hypertension and education level. The risk posed by decreasing educational attainment increased linearly and the linear trend was significant in women; however, no clear trend was seen in men. With regard to monthly income, women also showed a significant association with inverse trends for incident hypertension across all income levels. Compared with the high income group (≥2,000,000 KRW), the adjusted HR (95 % CI) for the low income group (<1,000,000 KRW) was 1.63 (1.25-2.12) (Model 1), and the association was significant even after adjustment for BMI (Model 2: HR = 1.62, 95 % CI = 1.25–2.11) and WC (Model 3: HR = 1.53, 95 % CI = 1.17–1.99). In men, monthly income was inversely associated with incident hypertension. Compared with the high income group, the adjusted HR (95 % CI) for the medium income was 0.76 (0.61–0.96) (Model 1), and the association was significant even after adjustment for BMI (Model 2: HR = 0.77, 95 % CI = 0.61–0.97) and WC (Model 3: HR = 0.77, 95 % CI = 0.61–0.96).

**Table 2:** Association of socioeconomic status with incident hypertension

	Education attainment			Monthly household income (×10^4^ KRW)		
	High (≥10 years)	Medium (7–9 years)	Low (0–6 years)	High (≥200)	Medium (100–199)	Low (<100)
	HR	HR (95 % CI)	HR (95 % CI)	HR	HR (95 % CI)	HR (95 % CI)
Men						
Model 1	1.00	1.15 (0.92–1.43)	1.08 (0.84–1.38)	1.00	0.76 (0.61–0.96)	1.11 (0.87–1.42)
Model 2	1.00	1.14 (0.92–1.42)	1.16 (0.91–1.49)	1.00	0.77 (0.61–0.97)	1.17 (0.92–1.50)
Model 3	1.00	1.12 (0.90–1.39)	1.15 (0.90–1.47)	1.00	0.77 (0.61–0.96)	1.13 (0.89–1.44)
Women						
Model 1	1.00	1.54 (1.16–2.06)	1.80 (1.36–2.38)	1.00	1.10 (0.83–1.45)	1.63 (1.25–2.12)
Model 2	1.00	1.52 (1.14–2.03)	1.75 (1.32–2.31)	1.00	1.12 (0.85–1.47)	1.62 (1.25–2.11)
Model 3	1.00	1.49 (1.12–1.99)	1.67 (1.26–2.21)	1.00	1.08 (0.82–1.43)	1.53 (1.17–1.99)

Abbreviation: *HR* hazard ratio; *KRW* South Korea won; Model 1 included age, smoking status, alcohol consumption, blood pressure level, and family history of hypertension for adjustment. Model 2 included the covariate in model 1 and body mass index (BMI). Model 3 included the covariate in model 1 and waist circumference (WC)

Table 3 shows the association between SES and hypertension in different strata of BMI (≥25 or <25) and WC (central obesity or not) after adjusting for confounding factors. For household income, men with obese BMI and medium income levels (HR = 0.72, 95 % CI = 0.52–1.00) were at a lower risk of incident hypertension than those with high income level. Those with central obesity and medium income levels (HR = 0.63, 95 % CI = 0.40–0.97) were also at a lower risk of incident hypertension than those with high income level. There was no significant association between education levels and SES in men.

**Table 3:** Association of socioeconomic status with incident hypertension, stratified by BMI and WC

		Education attainment (years)				Monthly household income (*10^4^ KRW)			
		High (≥10 years)	Medium (7–9 years)	Low (0–6 years)		High (≥200)	Medium (100–199)	Low (<100)	
		HR	HR (95 % CI)	HR (95 % CI)	*P* for trend	HR	HR (95 % CI)	HR (95 % CI)	*P* for trend
Men									
BMI (kg/m^2)^	≥25	1.00	1.06 (0.77–1.47)	0.94 (0.62–1.41)	0.722	1.00	0.72 (0.52–1.00)	0.96 (0.65–1.41)	0.829
	<25	1.00	1.25 (0.92–1.69)	1.28 (0.92–1.77)	0.155	1.00	0.83 (0.59–1.16)	1.37 (0.99–1.92)	0.061
Central Obesity	Yes	1.00	1.07 (0.71–1.62)	1.30 (0.83–2.05)	0.747	1.00	0.63 (0.40–0.97)	0.96 (0.62–1.50)	0.870
	No	1.00	1.19 (0.91–1.54)	1.06 (0.79–1.43)	0.201	1.00	0.81 (0.61–1.06)	1.19 (0.89–1.60)	0.235
Women									
Obesity	Yes	1.00	1.53 (1.02–2.29)	1.85 (1.24–2.75)	0.041	1.00	1.10 (0.76–1.61)	1.61 (1.12–2.31)	<0.001
	No	1.00	1.47 (0.97–2.23)	1.62 (1.08–2.41)	0.069	1.00	1.06 (0.70–1.60)	1.63 (1.10–2.40)	0.015
Central Obesity	Yes	1.00	1.54 (0.94–2.52)	1.63 (1.03–2.57)	0.037	1.00	1.12 (0.72–1.76)	1.42 (0.94–2.16)	0.004
	No	1.00	1.48 (1.03–2.12)	1.71 (1.17–2.49)	0.005	1.00	0.98 (0.69–1.41)	1.67 (1.18–2.37)	0.078

*HR* hazard ratio; *KRW* South Korea won; *BMI* body mass index; The central obesity was defined as WC ≥ 90 cm in men and WC ≥ 85 cm in women; Model included age, smoking status, alcohol consumption, blood pressure level, and family history of hypertension for adjustment

Among women with low education level, the adjusted HR for incidence of hypertension was 1.85 (95 % CI = 1.24–2.75) among those with obese BMI, 1.62 (95 % CI = 1.08–2.41) among those with non-obese BMI. 1.63 (95 % CI = 1.03–2.57) among those with central obesity, and 1.71 (95 % CI = 1.17–2.49) among those without central obesity. In addition, among women with low income level, the adjusted HR for incidence of hypertension was 1.61 (95 % CI = 1.12–2.31) among those with obese BMI, 1.63 (95 % CI = 1.10–2.40) among those with non-obese BMI. 1.42 (95 % CI = 0.94–2.16) among those with central obesity, and 1.67 (95 % CI = 1.18–2.37) among those without central obesity.

## Discussion

In this prospective study, the results showed gender differences in the relationship between SES and the incidence of hypertension in Korean adults. In women, both education level and household income were inversely associated with risk of hypertension, even after adjusting for potential confounders (Model 1), whereas in men positive association was found between household income and risk of hypertension. To our knowledge, no reported study has investigated associations between SES and the incidence of hypertension in Korea.

Several studies have demonstrated an association between SES and the prevalence of hypertension [7, 8, 13–15, 18, 19]. For example, in the Incidence of Hypertension in a French Working Population (IHPAF) Study, SES, measured by education level, was inversely associated with prevalence of hypertension in both men and women [7]. In data from the 2002–2003 Joint Canada/United States Survey of Health (JCUSH), household income showed a significant inverse linear relationship with hypertension prevalence [18]. In an Asian population, a study conducted in China reported an inverse association between educational attainment and prevalence [8]. A study of rural Vietnamese also showed an inverse association between educational attainment and hypertension prevalence in men, but not in women. Additionally, economic level was positively associated with hypertension in men, but inversely associated in women [19]. In Korea, only a few cross-sectional studies have been reported, with inconsistent results. Jo et al. found an inverse association between household income, but not education, and hypertension in both men and women in an age-adjusted model [13], and Cha et al. reported a higher prevalence of hypertension in individuals with low educational attainment, but found no association with household income [15]. However, studies with a prospective design are scarce [10–12]. In the First National Health and Nutrition Examination Survey (NHANSE I) Epidemiologic Follow-up Study (NHEFS) (1971–1984), education was found to be inversely associated with hypertension incidence among younger (aged 25–44 years) but not older (aged 45–64) non-Hispanic white men and women [10]. In the U.S. Women’s Health Study, education, but not income, was also inversely associated with incident hypertension [11]. In the Electricity Generating Authority of Thailand study, low educational attainment was reported to be associated with a higher incidence of hypertension during a 22-year follow-up period [12]. Our data on hypertension incidence were consistent with the findings of previous prospective studies.

An interesting finding of the present study is that the HRs for the relationship between SES and incident hypertension were slightly reduced (but remained significant) after additional adjustments for BMI (Model 2) or WC (Model 3) in women, whereas slightly (although non-significantly in education level) increased in men. In our study, SES was positively associated with obesity, but not central obesity in men. In women, education levels rather than a higher level of income had a greater effect on obesity and central obesity (data not shown). These findings were similar to those reported in prior research [6]. Above findings suggest that adiposity could affect the relationship between SES and risk of hypertension differently in men and women. Thus, to explore these issues, we also investigated the association between SES and hypertension into different strata of BMI and WC. In these analyses, we also found gender differences in the relationship between SES and hypertension incidence. In men, after stratification by BMI and WC, the positive associations of income level with hypertension incidence were significant only among participants with obese BMI or central obesity but the significance disappeared among participants without obese BMI or central obesity. However, in women, the inverse associations of both education and income levels with hypertension incidence were still significant after stratification by BMI and WC. Taking BMI or WC into account in multivariate analyses may result in partial masking of the relationship between SES and the incidence of hypertension.

This study found that the pattern of association between SES and risk of hypertension differed for men and women. The influence of low SES on hypertension was more prominent in women. While the reasons for the sex-related difference remain unclear, several plausible explanations that relate low SES to an increased risk of hypertension in women deserve consideration. For example, socioeconomic disparities may cause differing lifestyle behaviors, such as alcohol intake and smoking. Individuals with a lower SES tend to exhibit unhealthy lifestyle behaviors. Lower SES in women has been related to harmful alcohol consumption, cigarette smoking, and a lack of physical activity [20–22]. Furthermore, lower SES is related to less social support and poorer access to health care services, leading to less detection and treatment of hypertension and associated risk factors [23]. Finally, lower SES may be related to poor eating habits. Women with lower SES tend to eat fewer fruits and vegetables and choose a low-cost, high-fat, and energy-dense diet linked to obesity [24, 25].

Although the findings from this study show a relationship between SES and hypertension incidence, several limitations must be considered as well. First, we were unable to obtain comprehensive information on ‘conventional’ risk factors, such as salt intake, which are recognized as important risk factors for hypertension. Second, the relatively short (4-year) follow-up period may not provide an accurate estimate of the number of hypertension cases that developed. In the future, it may be desirable to evaluate the incidence of hypertension over a longer follow-up period. Finally, there is a possible bias arising from loss to follow-up and missing data on baseline risk variables.

## Conclusion

SES has a considerable impact on incidence of hypertension in Korean adults. Also, there were gender differences in the relationship between SES and hypertension. Because the prevalence of hypertension is high and the adverse cardiovascular consequences are frequently accompanied by untreated and uncontrolled hypertension, optimal BP control is important public health concern. Thus, a stratified approach for women of low socioeconomic status, especially those with low educational attainment, may be helpful for prevention and treatment of hypertension.
